# Distal Bile Duct Metastasis From Rectal Cancer: The Diagnostic Contribution of Intraductal Ultrasonography

**DOI:** 10.1002/deo2.70227

**Published:** 2025-10-15

**Authors:** Shinji Monoe, Ryo Nishio, Arihiro Nakano, Yu Yasue, Takahiro Yamashita, Hitoshi Iwata

**Affiliations:** ^1^ Department of Gastroenterology Nakatsugawa Municipal General Hospital Gifu Japan; ^2^ Department of Pathology Nakatsugawa Municipal General Hospital Gifu Japan

**Keywords:** bile duct, intraductal, metastasis, rectal cancer, sclerosing cholangitis

## Abstract

A 49‐year‐old male developed liver dysfunction during chemotherapy for rectal cancer located in the rectosigmoid region. Although magnetic resonance cholangiopancreatography initially indicated sclerosing cholangitis, endoscopic retrograde cholangiopancreatography and intraductal ultrasonography revealed multiple non‐contiguous intraductal masses. Histopathological analysis confirmed distal bile duct metastasis from rectal cancer, characterized by papillary intraductal lesions. The distribution and morphology of the tumors suggested implantation via bile flow or hematogenous dissemination through the peribiliary capillary plexus. Extrahepatic bile duct metastasis from colorectal cancer is exceptionally rare and poses significant diagnostic challenges. This report presents a rare case of distal bile duct metastasis detected during chemotherapy for rectal cancer, where endoscopic imaging was instrumental in establishing the diagnosis.

## 1 Introduction

Biliary strictures can result from malignant tumors such as primary cholangiocarcinoma and benign diseases such as primary sclerosing cholangitis (PSC) and IgG4‐related sclerosing cholangitis (IgG4‐SC), often complicating accurate diagnosis.

In the present rectal cancer case, a distal bile duct stricture developed in the rectosigmoid (Rs) region following liver dysfunction. Initial magnetic resonance cholangiopancreatography (MRCP) demonstrated irregular biliary strictures, raising concern for SC; however, subsequent endoscopic and histopathological evaluations confirmed bile duct metastasis from colorectal cancer. This underscores the diagnostic value of endoscopic assessment. We present clinically relevant insights into the differential diagnosis of bile duct strictures.

## 2 Case Report

A 49‐year‐old male was referred to our department after a positive fecal immunochemical test during routine colorectal cancer screening. Colonoscopy revealed a type 2 tumor in the rectal Rs region; biopsy confirmed adenocarcinoma. Contrast‐enhanced chest and abdominal computed tomography (CT) showed regional lymph node metastasis without evidence of distant spread. The patient underwent laparoscopic low anterior resection with D3 lymph node dissection and ileostomy formation. Histopathological analysis showed moderately differentiated adenocarcinoma (tub2). The carcinoma extended beyond the muscularis propria into the perirectal soft tissue with lymphatic, venous, and perineural invasion. Metastatic carcinoma was observed in 7/17 nodes (pN2b): 2/12 pericolic/perirectal (#251), 2/2 intermediate (#252), and 3/3 apical nodes at the inferior mesenteric artery root (#253). It was classified as pT3N2BM0 pStageIIIC following the UICC eighth edition.

One month postoperatively, rising serum carcinoembryonic antigen (CEA) levels prompted contrast‐enhanced CT, which identified enlarged inguinal lymph nodes. Biopsy confirmed metastatic adenocarcinoma. Chemotherapy with folinic acid, fluorouracil, and oxaliplatin (FOLFOX) plus bevacizumab (BEV) was initiated by the surgeon two months postoperatively.

At four months postoperatively, liver and bone metastases were detected. Since chemotherapy had recently begun, FOLFOX plus BEV was continued. After 22 cycles, multiple pulmonary metastases appeared at 15 months, prompting a regimen change to folinic acid, fluorouracil, and irinotecan plus BEV. Disease progression continued, and regorafenib was introduced at 18 months postoperatively.

At 19 months postoperatively, the patient developed liver dysfunction. *On initial assessment by the surgical oncology team*, MRCP showed strictures at the distal common bile duct and hepatic hilum, and *PSC/IgG4‐SC were considered in the differential diagnosis*. Serum IgG4 levels were not measured, and the patient was managed conservatively. At 20 months postoperatively, trifluridine/tipiracil hydrochloride was initiated due to severe hand–foot syndrome related to regorafenib.

At 21 months postoperatively, liver dysfunction worsened, and the patient was re‐evaluated. Blood tests (white blood cells, 6,700/µL; platelets, 305,000/µL; aspartate aminotransferase, 156 U/L; alanine aminotransferase, 69 U/L; alkaline phosphatase, 302 U/L; gamma‐glutamyl transpeptidase, 302 U/L; total bilirubin, 0.9 mg/dL; and C‐reactive protein, 7.08 mg/dL) indicated elevated hepatobiliary enzymes. Immunologic markers (immunoglobulin G, 1,064 mg/dL; immunoglobulin G subclass 4, 59 mg/dL; antinuclear antibody titer, 1:40; and negative anti‐mitochondrial M2 antibody [<1.5]) suggested no significant autoimmune activity.

**FIGURE 1:**
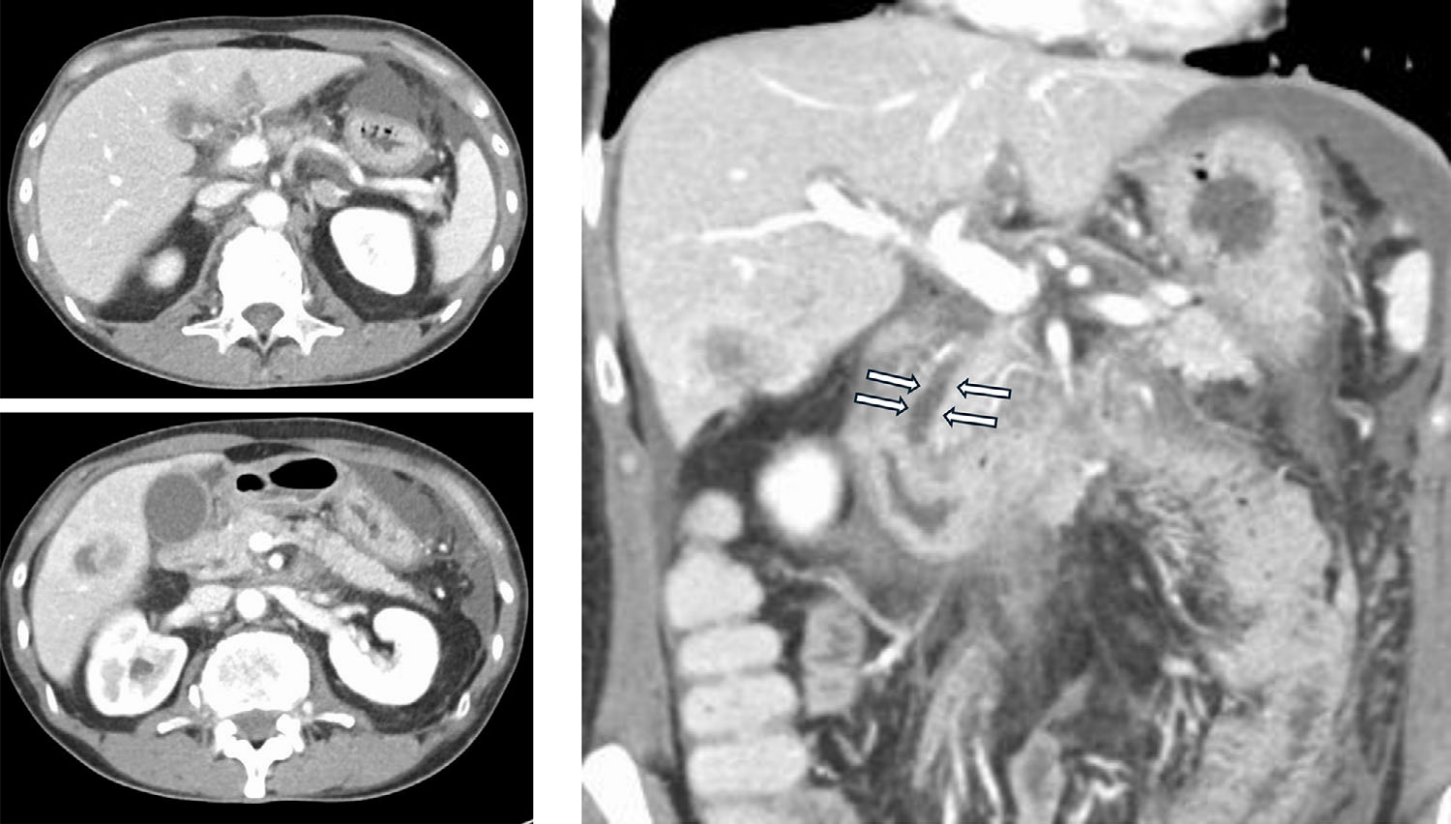
Contrast‐enhanced computed tomography (CT) revealed multiple liver metastases; no biliary ductal dilatation or intraductal masses were seen.

**FIGURE 2:**
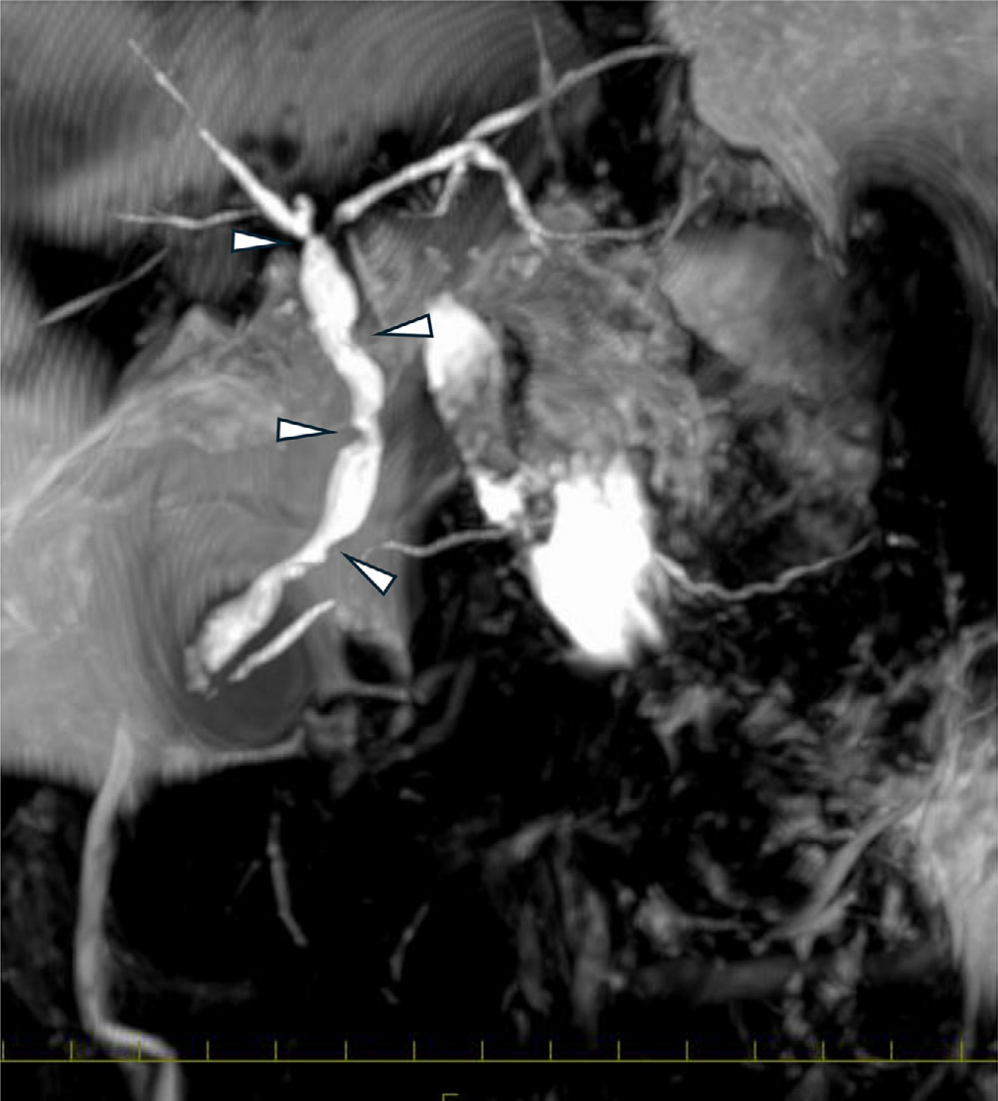
Magnetic resonance cholangiopancreatography (MRCP) demonstrated irregular strictures in the hilar and distal bile ducts. The intrahepatic bile ducts exhibited a pruning‐like appearance. A distinct punch‐out‐like stricture with intraluminal protrusion was observed in the distal bile duct.

Contrast‐enhanced abdominal CT did not reveal clear abnormalities (Figure 1); however, MRCP demonstrated irregular strictures in the hilar and distal bile ducts. Intrahepatic bile ducts exhibited a pruned tree pattern, while the distal bile duct displayed a punch‐out‐like stricture with a suspected intraluminal mass (Figure 2). Although the pruned‐tree changes were limited, the initial radiology report included SC as a differential diagnosis. At that time, the patient was referred to our department for further evaluation. These findings suggested SC in the intrahepatic ducts and a tumor‐like lesion in the distal bile duct.

**FIGURE 3:**
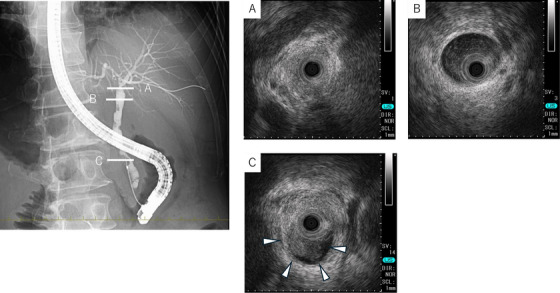
Endoscopic retrograde cholangiopancreatography (ERCP) revealed multiple intraductal protrusions without frayed, beaded, or diverticulum‐like features. Biopsies were taken from the sites labeled A and C. Representative intraductal ultrasonography (IDUS) images are shown: (A) Hilar bile duct: Circumferential luminal narrowing was evident, with disruption of the outer hyperechoic band and loss of the normal layered architecture. (B) Non‐stenotic region: The bile duct wall was smooth, with preservation of both the layered structure and the outer hyperechoic band. (C) Distal bile duct: A protruding lesion was visualized at the 8 o'clock position.

Endoscopic retrograde cholangiopancreatography (ERCP) identified multiple irregular papillary intraductal tumors and a hilar stricture; however, the MRCP features of SC were not replicated. Intraductal ultrasonography (IDUS) revealed well‐demarcated but irregular tumors with heterogeneous hypoechogenicity, loss of the outer high‐echoic layer, and bile duct wall disruption. No dilation or strictures were observed outside the tumor sites, and ductal wall architecture was preserved (Figure 3).

**FIGURE 4:**
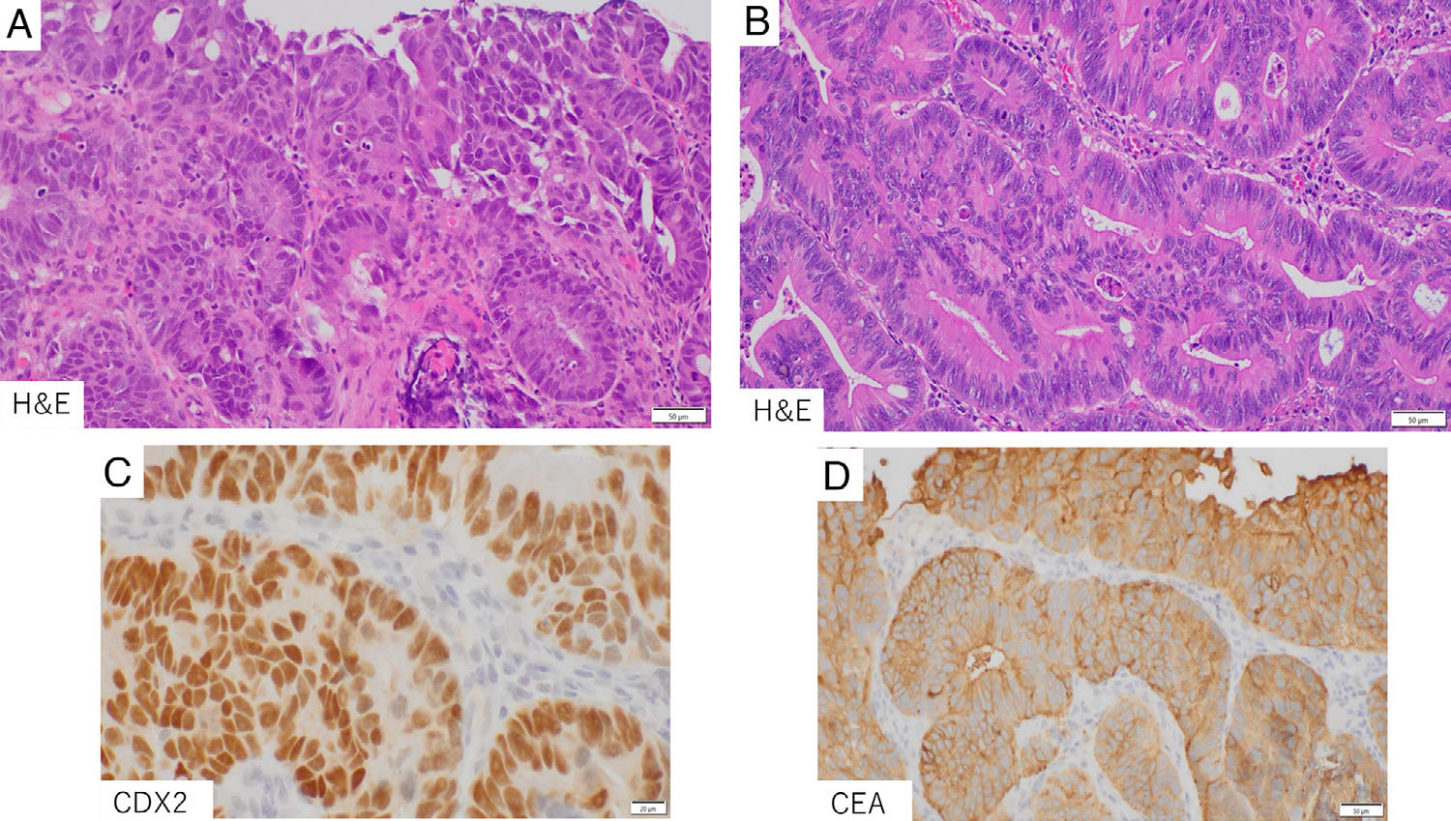
Histopathological analysis of the bile duct lesion and primary colorectal tumor: (A) Bile duct lesion, hematoxylin and eosin (H&E): well to moderately differentiated tubular adenocarcinoma confined to the mucosa. The tumor cell morphology was similar to that of the previously resected colorectal carcinoma. (B) Primary colorectal tumor (H&E), a tubular adenocarcinoma architecture, with atypical glandular epithelium forming tubular to fused glands. (C) CDX2 immunostaining of the bile duct lesion: nuclear positive. (D) CEA immunostaining of the bile duct lesion: cytoplasmic positive.

Biopsy of the bile duct lesion revealed tubular adenocarcinoma replacing the native biliary epithelium in foci. Owing to fragmentation/crush artifact, assessment of invasion depth and stromal reaction was limited. Immunohistochemistry showed nuclear positivity for CDX2, cytoplasmic positivity for CEA (Figure 4A,C,D), and was negative for carbohydrate antigen 19‐9 (CA19‐9). These findings mirrored the histologic and morphological features of the primary rectal cancer (Figure 4B). No additional primary malignancies were detected on abdominal CT or upper gastrointestinal endoscopy, confirming bile duct metastasis from rectal cancer.

To alleviate liver dysfunction due to biliary obstruction, a plastic stent was endoscopically inserted, leading to improved hepatic function and subsequent discharge. Chemotherapy was discontinued, and palliative care was selected. At 23 months postoperatively, the patient developed coronavirus disease 2019, which led to rapid clinical deterioration and death.

## 3 Discussion

Biliary strictures caused by metastases may mimic primary bile duct malignancies or benign cholangiopathies, such as cholangiocarcinoma, IgG4‐SC, and PSC. Here, MRCP initially suggested SC, with a pruned tree appearance in the intrahepatic bile ducts and a punch‐out‐like lesion in the distal bile duct. However, serum IgG4 levels were within normal range, and ERCP with IDUS demonstrated multiple papillary intraductal masses with disrupted bile duct wall layering, raising concern for malignancy.

IDUS is a valuable tool for assessing bile duct lesions and plays a key role in distinguishing cholangiocarcinoma from IgG4‐SC, with diagnostic clues such as the symmetry of wall thickening and preservation of layered structure [1, 2]. In IgG4‐SC, uniform concentric thickening typically extends beyond the stenotic segment into adjacent non‐stenotic areas. Contrarily, cholangiocarcinoma usually presents with localized, asymmetric thickening and loss of normal layering.

A bile duct wall thickness cutoff of 1 mm differentiates malignant from benign lesions, with 95% sensitivity, 90.9% specificity, and 93.5% diagnostic accuracy [1]. In PSC, IDUS typically reveals an irregular inner margin, diverticulum‐like outpouchings, and disrupted wall layering, distinguishing it from IgG4‐SC [2].

In this case, IDUS demonstrated localized, irregular thickening with loss of normal layering. No diverticulum‐like protrusions were identified, and the bile duct wall outside the lesion appeared smooth and measured <1 mm in thickness. These findings, combined with the clinical course, supported the suspicion of malignancy and warranted a biopsy. EUS could have complemented the work‐up by assessing extramural extension and regional lymph nodes; however, it was not performed in this case.

Although IDUS characteristics of metastatic bile duct tumors have been infrequently described [3], this case suggests that attention to differences from benign biliary strictures may aid in the differential diagnosis. The metastatic pathways to the bile duct are not fully understood; however, several mechanisms have been proposed, including (1) hematogenous spread via the peribiliary capillary plexus (PBCP) [4]; (2) tumor cell implantation via bile flow [5]; (3) procedure‐related seeding from biliary interventions [6]; and (4) lymphatic dissemination from rectal cancer or invasion from existing metastatic lesions. [7]

Here, multiple intraductal protrusions and strictures were present from the hepatic hilum to the intrapancreatic bile duct. The non‐contiguous and multidirectional nature of these lesions suggested bile flow–mediated implantation or hematogenous spread through the PBCP.

Implantation refers to the detachment of tumor cells into the bile, which are subsequently carried downstream, adherence to the epithelium, and proliferation. Ofuchi et al. [8] proposed this mechanism in postoperative, non‐contiguous lesions spanning from the hepatic hilum to the cystic duct. Similarly, Okano et al. [9] reported histologic confirmation of bile duct invasion (42%) with liver metastases from colorectal cancer, indicating that extension into the intrahepatic bile ducts is frequent.

Here, it is plausible that intrahepatic metastatic lesions invaded the bile ducts, releasing tumor cells into the bile, leading to distal duct seeding. This implantation mechanism aligns with the observed non‐contiguous lesion distribution.

The PBCP, a fine capillary network beneath the biliary epithelium, may also serve as a route for tumor infiltration into the bile duct wall. Through this pathway, intraductal tumors may form without visible mucosal disruption. In this case, IDUS showed no significant abnormalities in the bile duct wall outside the protrusions, and the tumor lesions were discontinuously distributed, supporting localized invasion via the PBCP.

In conclusion, although uncommon, bile duct metastasis from colorectal cancer should be considered in the differential diagnosis of biliary strictures, particularly in patients with a history of colorectal malignancy. A thorough diagnostic strategy—including imaging, endoscopic assessment, histopathology, and immunohistochemistry—is critical for distinguishing metastatic disease from primary biliary disorders and guiding appropriate management.

## Author Contributions

**Ryo Nishio**, **Arihiro Nakano**, and **Yu Yasue**: resources. **Takahiro Yamashita**: investigation. **Ryo Nishio**: supervision. **Arihiro Nakano**, **Yu Yasue**, and **Takahiro Yamashita**: writing—review & editing. **Hitoshi Iwata**: investigation and writing—review & editing. All authors have read and approved the final version of the manuscript.

## Ethics Statement

**Approval of the research protocol by an Institutional Review Board**: N/A

## Informed Consent

Informed consent for this case report was obtained from the patient.

## Conflicts of Interest

The authors declare no conflicts of interest.
